# Semantic and pragmatic precision in conversational AI systems

**DOI:** 10.3389/frai.2023.896729

**Published:** 2023-03-30

**Authors:** Harry Bunt, Volha Petukhova

**Affiliations:** ^1^Department of Cognitive Science and Artificial Intelligence, Tilburg University, Tilburg, Netherlands; ^2^Spoken Language Systems Group, Saarland University, Saarbrücken, Germany

**Keywords:** conversational AI agents, dialog acts, dialog modeling, semantically and pragmatically motivated interaction analysis, human-agent data collection

## Abstract

For a conversational agent, to display intelligent interactive behavior implies the ability to respond to the user's intentions and expectations with correct, consistent and relevant actions with appropriate form and content in a timely fashion. In this paper, we present a data-driven analytical approach to embed intelligence into a conversational AI agent. The method requires a certain amount of (ideally) authentic conversational data, which is transformed in a meaningful way to support intelligent dialog modeling and the design of intelligent conversational agents. These transformations rely on the ISO 24617-2 dialog act annotation standard, and are specified in the Dialogue Act Markup Language (DiAML), extended with plug-ins for articulate representations of domain-specific semantic content and customized communicative functionality. ISO 24617-2 is shown to enable systematic in-depth interaction analysis and to facilitate the collection of conversational data of sufficient quality and quantity of instances of interaction phenomena. The paper provides the theoretical and methodological background of extending the ISO standard and DiAML specifications for use in interaction analysis and conversational AI agent design. The expert-assisted design methodology is introduced, with example applications in the healthcare domain, and is validated in human-agent conversational data collection experiments.

## 1. Introduction

A conversational AI system can engage in an intelligent conversation with a human user, producing communicative behavior that is pragmatically and semantically adequate. Pragmatic adequacy means responding to the user's intentions and expectations in a functionally meaningful way and doing so in a form that is appropriate in the given context. Semantic adequacy means that the system responds with semantic content that is correct, consistent and relevant given the semantic structure of the user's intentions and information state.

Conversational agents come in a range of forms and under different names. As observed in Laranjo et al.'s (2018) survey of the field, there is no general consensus regarding the definitions of “conversational agents,” “dialog systems,” “embodied conversational agents,” “smart conversational interfaces,” or “chatbots.” Systems of all these denominations are able to engage in some form of dialog with a user, making use of natural language both for their input and their output. They can be roughly divided into task-oriented and general-purpose ones. Task-oriented agents can operate in and communicate about tasks of a certain kind, which they are knowledgeable about either by design or through training. Question-answering systems exemplify this type of agent. Depending on the quality (a) of their language understanding, and (b) of their domain knowledge, such systems can reach a considerable degree of semantic precision, though only for the closed domain they have knowledge about; pragmatically they tend to be poor, being mostly limited to communicate in the form of question-answer sequences, and lacking the abilities to interpret and produce feedback and interaction management actions, which are largely responsible for the smoothness of human conversational interaction. As the survey by Zaib et al. (2021) concludes, from a conversational point of view question-answering systems are still in their infancy.

General-purpose conversational agents are exemplified by open-domain chatbots, which have the ability to engage in small talk and casual conversation on any topic. Their behavior is only superficially somewhat human-like and “conversational,” exhibiting verbal fluency and lexical variability, and much of the time little intelligence due to insufficiently deep semantic and pragmatic understanding, plus a lack of common-sense knowledge. The lack of precision in semantic and pragmatic understanding precludes an intelligent form of dialog management. Instead, techniques like slot-filling and lists of frequently asked questions with pre-cooked answers are often used to produce the system's interactive behavior.

Chatbots are often designed to act as active listeners by generating simple feedback responses like “I got it” or asking encouraging Eliza-style questions like “Do you want to tell me more?”, not requiring the bot to fully understand the user's response for advancing the conversation. More advanced chatbots based on machine learning and neural models, such as Meena (Adiwardana et al., 2020) show more human-like behavior, which is generated by predicting the next utterance in a conversation given previous utterances, i.e., mapping (encoding-decoding) from sequence to sequence, element by element learned from large dialog corpora and other texts. However, systems of this type typically are unable to discuss articulate semantic content. They have been observed to suffer from repetitiveness, semantic inconsistency, and pragmatic irrelevance, and present only a rough approximation of what would be expected of an intelligent conversational agent.

In this paper we argue, supported by experiences in the analysis and design of intelligent cooperative multimodal systems, that pragmatic and semantic precision in the behavior of conversational agents can benefit from the use of a powerful representation and interchange language. We consider for this purpose the Dialogue Act Markup Language (DiAML), defined as part of ISO standard 24617-2 (ISO, 2020) for dialog annotation, extended with plug-ins for (a) the representation of semantic content; (b) the addition of domain-specific communicative functions; and (c) more articulate representations of affect. Affect can be pragmatically important for an AI agent, for example, a digital nurse, observing a patient to be in a worrisome state, may not be truly distressed or alarmed, but should act *as if* they are alarmed, and perform the necessary actions with appropriate haste. Moreover, even though they are not capable of experiencing human emotions and feelings, they need to recognize and to some degree understand such states in order to be able to interact successfully with the patient. Using the healthcare domain for making things concrete, we aim to show, on the basis of a detailed analysis of recorded conversations, how the extended representation framework facilitates (1) in-depth interaction analysis; (2) interactive data collection; and (3) the design of intelligent conversational agents by domain experts.

This paper is organized as follows. Section 2 discusses a number of basic functional requirements of an intelligent conversational agent, and how these requirements translate to properties of a data representation and interchange language. The relevant features of DiAML are summarized from this perspective. Section 3 discusses the use of DiAML representations and ISO 24617-2 concepts to support (a) interaction analysis, in particular in the medical domain; (b) expert-assisted authoring of AI agents; and (c) interactive data collection with simulated conversational AI agents. Section 4 focuses on the adaptation and customization of the ISO 24617-2 framework for the healthcare domain, regarding both the representation of medical content and the inclusion of communicative functions specific for medical consultations. Section 5 concludes the paper with observations on the experiences reported in previous sections, and perspectives of the proposed approach for data-driven design of future generations of intelligent conversational agents.

## 2. Semantic and pragmatic precision

### 2.1. Functional decomposition and representation in a conversational agent

The intelligence of a conversational agent's behavior can be boosted by the use of semantically and pragmatically analyzed data rather than raw verbal data, enabling it to generate not just certain word sequences but certain types of purposeful communicative action, based on the use of intelligent interactive strategies, external knowledge sources, and simulation of human reasoning. The semantic and pragmatic enrichments of the analyzed conversational data should be such that they provide effective support for performing the following functions, which an intelligent conversational agent should somehow perform, regardless of its architecture.

1. Input recognition: The determination of what the user types, says or gestures, supported by contextual knowledge and expectations about user behavior.
2. Intent recognition: the recognition of what the conversational partner is trying to achieve by what (s)he types, says, or gestures.
3. Determination of semantic content. For deep semantic understanding, this includes relating the meaning of lexical units to a domain ontology, computing local aspects of sentence meaning, and resolution of anaphora and other contextual meaning aspects.
4. Identification of pragmatic and semantic relations with previous utterances (such as question—answer, statement—correction, and inform—elaboration).
5. Recognition of affect (emotions, empathy, sentiment,...).
6. Maintenance of a representation of the dialog state. Understanding the user's input creates a new situation for the agent to respond to, context awareness being the key to avoiding redundancies and inconsistencies. This new situation forms an information state, of which the dialog history is a part.
7. Generation of a functionally relevant and semantically precise continuation of the dialog, given the understanding of the user's last utterance, the further dialog history and other properties of the dynamic context. This function is the heart of dialog management.
8. Output formulation: Construction of an appropriate form of the generated functionally and semantically determined continuation, given the dialog history and the communicative setting, in particular the use of interactive modalities.

Of these functions, input recognition can be supported by making use of the dialog history and expected continuations of an ongoing dialog, by representing user contributions (as well as system contributions) not just as raw text or speech, but as *dialog acts*, carrying certain intentions and assumptions. Since DiAML was designed to describe dialog acts (DAs), it can be put to good use in the representation of such knowledge and expectations. Intent recognition can be captured by describing the *communicative functions* of user inputs. A range of proposals has been put forward for this purpose, the most recent and most detailed ones being the DIT++ and ISO 24617-2 taxonomies, which are close relatives (see Section 3). DiAML represents the communicative functions of utterances using either of these taxonomies (or other sets of functions, for that matter), and is thus eminently suitable as an interchange language for the results of intent recognition, as well as for annotating training data for this purpose.

For example, the request in (1a) can be represented in DiAML as shown in XML format in (1b)^1^ and in a simpler, better human-readable format^2^ as the nested triple in (1c).

(1) a. 1. User1: Wait a moment please.
b. < dialogAct xml:id="a15" target="#m9" sender="user1" addressee="sys" dimension="timeManagement"communicativeFunction="request"/ >
c. 〈a15, m9, 〈User1, Sys, TimeM, Request〉〉

In the semantic-pragmatic framework which underlies the ISO and DIT++ taxonomies, functional specifications of dialog acts, like in (1b) or in (1c) have a semantic interpretation as functions which, applied to a semantic content, form an update operation on dialog states (see, Bunt, 2014). This update semantics makes DiAML ideal for using the results of intent recognition as inputs to the function of maintaining a representation of the dialog state. However, the specification of this update semantics requires a representation of semantic content to be available. Since the ISO standard was designed to be applicable across domains, languages and platforms, it does not include the domain-specific concepts needed for semantic content representation. To overcome this limitation, the mechanism of *plug-ins* has been introduced (Bunt, 2019); this mechanism is discussed in Section 2.3. With domain-specific content plug-ins, DiAML is able to support the function of semantic content determination with the desired precision.

For representing the data produced by the generation of functionally relevant dialog continuations, the expressivity of DiAML illustrated in (1) can be effectively used, as shown in the system response (2) to the user's request (1a); an output form for this dialog act would be “Certainly!”.

(2) a. < dialogAct xml:id="a16" target="#m10" sender="sys" addressee="user1" dimension="timeManagement"communicativeFunction="acceptRequest"qualifier="certain"/ > < functionalDependence dact="#a16"functAntecedent="#a15"/ >
b. 〈a16, m10, 〈Sys, User1, TimeM, 〈AcceptRequest, a15〉, certain〉〉

Other DA information that is represented in DiAML includes semantic dependence relations (like question—answer), pragmatic relations (like inform—elaborate), and qualifiers for uncertainty, conditionality, and affect; this makes DiAML also suitable for representing the output of the corresponding subfunctions of input interpretation. Besides plug-ins for semantic content representation, which are discussed in Section 2.3, plug-ins have also been defined for indicating emotions, inspired by EmotionML (Burkhardt et al., 2017), which make DiAML suitable for representing results of affect recognition. Moreover, extending the ISO 24617-2 repository of communicative functions with a plug-in that defines additional, domain-specific DA types, gives DiAML the expressive power to specify dialog continuations with appropriate pragmatic precision.

We will show, based on a detailed analysis of recorded doctor-patient consultations and simulated human-agent therapy planning negotiations, how functional aspects of dialog act specification can be tailored to a particular use case and enriched with domain-specific semantic content. The extended ISO 24617-2 repository of communicative functions and the semantically enriched DiAML representations have proven to facilitate (1) in-depth interaction analysis; (2) interactive data collection; and (3) the design of intelligent conversational agents by domain experts. These use cases are discussed in Section 3. The rest of this section provides a brief summary of DiAML and discusses the use of plug-ins.

### 2.2. ISO 24617-2

The ISO 24617-2 standard for dialog annotation is based on the analytical framework of dialog act theory (Bunt, 1999, 2000, 2011), and has as its centerpiece a multidimensional taxonomy of communicative functions, based on the DIT++ taxonomy (Bunt, 2009). A starting point of DIT is that participation in a dialog involves a range of communicative activities beyond those strictly related to performing a certain task or activity that motivates the dialog. The term “dimension” was introduced to refer to the different types of communicative activity, and the following 11 dimensions are distinguished, which are “orthogonal” in the sense that the function which a stretch of communicative behavior may have in one dimension is independent of its functions in other dimensions:

1. *Task:* dialog acts that move the task or activity forward which motivates the dialog;
2. *Task Management*: dialog acts discussing how to perform a given task;
3. *Auto-Feedback*: dialog acts providing information about the processing by the current speaker of previous utterances by another speaker;
4. *Allo-Feedback*: dialog acts providing or eliciting information about the processing by the addressee of previous utterances by the current speaker or another speaker;
5. *Turn Management*: activities for obtaining, keeping, releasing, or assigning the right to speak;
6. *Contact Management*: activities for establishing, checking, and maintaining contact;
7. *Time Management*: acts for managing the use of time in the interaction;
8. *Discourse Structuring*: dialog acts dealing with topic management, opening and closing (sub)dialogs, or otherwise structuring the dialog;
9. *Own Communication Management*: actions by the speaker to edit his current contribution;
10. *Partner Communication Management*: actions to edit a contribution of another current speaker;
11. *Social Obligations Management*: dialog acts that take care of social conventions such as greeting, introducing oneself, apologizing, and thanking.

In addition to communicative functions, DIT defines the following four aspects of dialog acts:

- *Qualifiers*, for expressing that a dialog act is performed conditionally, with uncertainty, or with a certain sentiment.
- *Functional dependence relations*, which link a dialog act to other dialog acts on which they depend for their semantic content, e.g., for indicating which question is answered by a given answer. This is the case for all dialog acts that are responsive in nature, such as Answer, Confirmation, Disagreement, Accept Apology, and Decline Offer.
- *Feedback dependence relations*, which link a feedback act to the dialog segment that it provides or elicits feedback about.
- *Rhetorical relations*, which indicate semantic or pragmatic relations between dialog acts, e.g., that one dialog act motivates the performance of another dialog act.

The ISO 24617-2 taxonomy consists of 64 communicative functions, some of which are specific for a particular dimension; for example, Turn Take is specific for Turn Management; Stalling for Time Management, and Self-Correction for Own Communication Management. Other functions, including all types of question, statement, and answer, as well as all commissive and directive functions, can be used in any dimension. These are called “general-purpose” functions, as opposed to “dimension-specific” ones. Table 1 shows the ISO inventory of communicative functions and dimensions.

**Table 1:** ISO 24617-2 communicative functions.

**General-purpose**	**Dimension-specific communicative functions**	
**Communicative functions**	**Function**	**Dimension**
*Information providing*	Auto positive	Auto-feedback
Inform	Auto negative	
Agreement	Allo positive	Allo-feedback
Disagreement	Allo negative	
Correction	Feedback elicitation	
Answer	Stalling	Time management
Confirm	Pausing	
Disconfirm	Turn take	Turn management
*Information seeking*	Turn accept	
Question	Turn grab	
Set question	Turn keep	
Propositional question	Turn release	
Check question	Turn assign	
Choice question	Contact check	Contact management
Test question	Contact indication	
*Commissives*	Self-error	Own communication management
Offer	Retraction	
Promise	Self-correction	
Address request	Completion	Partner communication management
Decline request	Correct misspeaking	
Accept request	Interaction structuring	Discourse structuring
Address suggest	Opening	
Accept suggest	Topic shift	
Decline suggest	Init-greeting	Social obligations management
*Directives*	Return greeting	
Request	Init-self-introduction	
Instruct	Return self-introduction	
Address offer	Apology	
Accept offer	Accept apology	
Decline offer	Compliment	
Suggest	Congratulation	
	Sympathy expression	
	Thanking	
	Accept thanking	
	Init-goodbye	
	Return goodbye	

### 2.3. Plug-in annotation schemes and interfaces

A plug-in annotation scheme is an add-on to a host annotation scheme. According to the principles of semantic annotation as laid down in ISO standard 24617-6 (ISO, 2016), a semantic annotation scheme has a three-part definition, consisting of (1) an abstract syntax which specifies the possible annotation structures at a conceptual level, in the form of set-theoretical structures, such as pairs and triples of concepts; (2) a semantics which specifies the meaning of annotation structures; (3) a concrete syntax which specifies a representation format for annotation structures (for example using XML).

Formally, the definition of an annotation scheme is thus a triple

(3) *X* = 〈*S*_*A*_, *S*_*C*_, *S*_*m*_〉

formed by specifications of an abstract syntax, a concrete syntax, and a semantics. Augmenting a host annotation scheme with a “plug-in” means augmentations at all three levels: abstract syntax, concrete syntax, and semantics. To make this possible, the specification of a plug-in includes specifications at all three levels; it has the three-part structure of (3). In addition, a *plug-in interface* is required, which specifies how the annotation structures of the host and the plug-in can be linked (see below).

The degree of detail in which semantic content is best represented depends on the application domain. For some domains a simple representation as a list of attribute-value pairs may be adequate; for others a representation in terms of events with their participants, time and place may be more appropriate; for semantically more complex applications it may be necessary to take general aspects of natural language utterance meaning into account, including phenomena of quantification, modification, and subordination.

The use of a semantic content plug-in *P*_*c*_ for the host annotation scheme *X*_*h*_ requires a plug-in interface _*h*_*Y*_*c*_, which has again the three-part structure of (3). The abstract syntax component of the interface introduces the content link structure as a pair 〈*a, c*〉 consisting of a dialog act structure (“*a*”) and a content structure (“*c*”); the concrete syntax specifies its XML encoding using the < contentLink> element, and the semantics specifies its meaning as the application of the interpretation function *I*_*h*_(*a*), defined by the semantics of the host annotation scheme, to the argument *I*_*c*_(*c*), defined by the plug-in semantics. This semantics reflects the dialog act theory of DIT, according to which the semantics of a full-blown DA is an update operation obtained by applying the semantics of the communicative function to its semantic content.

The host annotation system *X*_*h*_ together with the content plug-in *P*_*c*_ and the interface _*h*_*Y*_*c*_ forms an extended annotation scheme *X*_*h*+*c*_ defined by the unions of the components that make up the three parts of the host, plug-in, and interface schemes.

A simple plug-in for representing semantic content as a list of attribute-value pairs could for example be useful in a travel planning domain where a journey can be described by specifying, e.g., departure place, destination, travel date, etc. In such a context, the semantic content of the utterance “I'd like to leave around ten in the morning”, could be annotated as in (4b):

(4) a. I'd like to leave around ten in the morning (= markable *m*1)b. < avContent xml:id="c1" target="#m1"attribute="departureTime" value="10:00"/ >

To link an AV-content representation to DA representations, the XML element < contentLink>, defined in the interface _*h*_*Y*_*AV*_, can be used to obtain representations of the form (5).

(5) < dialogAct xml:id="da1" target="#m1" speaker="#s" addressee="#a" dimension="task" communicativeFunction="inform"/ > < avContent xml:id="c1" target="#m1"attribute="departureTime" value="10:00"/ > < contentLink dialAct="#da1" content="#c1"/ >

The use of an explicit link between the functional aspects of a dialog act and its semantic content allows the use of alternative plug-ins for content representation, and offers the possibility to customize it.

More details and discussion about plug-ins and plug-in interfaces can be found in Bunt (2019) and at http://dit.uvt.nl.

## 3. Semantic and pragmatic modeling with ISO 24617-2

The intelligence of a conversational agent's behavior can be boosted by the use of semantically and pragmatically analyzed data instead of raw verbal data. dialog data annotated with DA information can form the basis for design and training of conversational agents, personalized recommendations and interventions. Conversational agents can improve their performance if they generate not just certain word sequences but certain types of dialog acts, based on the application of intelligent interactive strategies and simulation of human reasoning (including, e.g., theory-of-mind skills). What types, where and how frequently specific dialog acts should be generated, is best estimated on the basis of analyzed real interactions and testing in simulated interactions. In the next sections, we show how the ISO-24617 dialog annotation framework facilitates three important use cases: (1) interaction analysis for agent design; (2) expert-assisted AI agent authoring; and (3) interactive in-domain annotated data generation. Given the tremendous and steadily growing interest, both academic and industrial, in conversational AI applications in healthcare settings (Laranjo et al., 2018), this domain is in focus here.

### 3.1. Interaction analysis

Systematic and comprehensive interaction analysis is often used for obtaining a satisfactory degree of understanding of human interactive behavior, as a basis for the specification of mechanisms of natural dialog to be incorporated into a conversational system. Such analysis typically involves annotation with dialog act information, for which annotation schemes have been developed that are useful both for empirical studies of general and task-related conversational phenomena, and for data-driven design of interactive systems. The objectives of interaction analysis can be generic, such as those of the Roter Interaction Analysis System (RIAS, Roter and Larson, 2002) or specific to a particular type of interaction, such as ISBAR for medical handovers (Spooner et al., 2018). They can also be specific to a particular aspect of communication, such as OPTION5 and OPTION12 for shared decision making (Elwyn et al., 2003), or specific to an element of communicative behavior such as emotions, like the Verona Coding Definition of Emotional Sequences (VR-CoDES) (Del Piccolo et al., 2011).

Publicly available dialog corpora containing real doctor-patient interactions are rare, for reasons of privacy and data security. The corpus considered in this study is rather small, but comprises authentic patient-doctor interactions from the Edinburgh-based patient-clinician communication project VICO.^3^ The VICO corpus contains 30 transcriptions of routine video consultations over the internet between patients and primary care clinicians, with a total duration of 4 h 35 min, and about 63,000 tokens. The average duration of a dialog is 8.5 min.

VICO dialogs were manually re-segmented and annotated with ISO 24617-2 dialog acts and independently with the RIAS categories by two trained annotators who were not medical experts and who were novice users of the RIAS scheme. Inter-coder agreement in terms of Cohen's kappa was moderate both for RIAS and for ISO (on average κ = 0.52 and 0.58, respectively). The annotations were compared and mapped. RIAS categories that were more specific and captured the utterance meaning more accurately, or that were not defined in the ISO taxonomy, were mapped to proposed elements extending the ISO scheme, see Section 4.

The resulting annotated dataset comprises 12,877 functional segments, 54.5% of which produced by the doctor and 45.5% by the patient. Table 2 shows the distribution of annotated dialog acts across the ISO dimensions with an extra dimension proposed in Petukhova and Bunt (2020). The analysis shows that the majority of functional segments has a function in the **Task** dimension. Doctors more often request information (85.6%) and give instructions (*counseling* actions to be undertaken by the patient, 71.9%), and patients respond to doctors' questions (68.2%), rarely ask their own (14.4%), and express commitment to perform requested actions following doctor's instructions (56.8%).

**Table 2:** Distribution of functional segments across dimensions produced by the doctor and the patient, in terms of relative frequency (in %).

**Dimension**	**Functional segments (in %)**		
		**ALL**	**From those**	
				**Doctor**	**Patient**
Task/activity	39.4	44.5	55.5
Task management	2.1	46.3	53.7
Auto feedback	16.4	72.2	27.8
Allo feedback	2.9	30.4	69.6
Discourse structuring	0.9	61.1	38.9
Own communication management	15.5	58.1	41.9
Social obligations management	3.6	60.0	40.0
Interpersonal relation management	3.5	62.7	37.3
Turn management	21.0	61.4	38.6
Time management	14.6	60.5	39.5

In medical interactions, it is important that the doctor not only listens actively but also shows a genuine interest and understanding of the patient's behavior, repeating information revealed, rephrasing previously asked questions or provided instructions, confirming or checking for understanding, consistency and validation of the information exchanged. **Auto-Feedback** acts are therefore more frequently observed as performed by the doctor than by the patient (72.2 vs. 27.8%). In the case of **Allo-Feedback** acts, the situation is the opposite (30.4% is produced by the doctor vs. 69.6% by the patient). Patient's Allo-Feedback acts are responses to doctor's verification and validation efforts and concern confirmation or correction of doctor's understanding and consistency beliefs.

Concerning the Interaction Management functions, **Turn Management** and **Time Management** acts are frequently observed (21.0 and 14.6%, respectively). These acts are more frequently performed by the doctor, executing control over the interaction. **Own Communication Management** acts occur quite frequently (15.5%), revealing difficulties that the participants encounter in expressing themselves.

Table 3 provides a more detailed overview of dialog acts and their distributions for two important dimensions: Task and **Interpersonal Relations Management** (IRM), an extension to the ISO set of dimensions for medical dialogs introduced by Petukhova and Bunt (2020). IRM acts aim at developing and maintaining a good doctor-patient relationship. This category comprises actions in order to (1) define the nature of the relationship; (2) communicate interest, respect, support, and empathy; (3) recognize and resolve relational barriers to patient-provider communication; and (4) elicit the patient's perspective. dialog acts are observed expressing worry and concern, reassurance and empathy. Doctors also encourage patients to ask questions, express their attitudes, preferences, concerns, fears, and opinions, i.e., motivate patients' self-disclosure.

**Table 3:** Distribution of doctor and patient task-related and interpersonal relations management dialog acts, in terms of relative frequency (in %).

**Communicative Function**	**Functional segments (in %)**		
	**ALL**	**From those**	
			**Doctor**	**Patient**
**Task-related exchange**			
-Info-seeking	13.7	85.6	14.4
-Info-providing	67.8	31.8	68.2
-Directives	8.4	71.9	28.1
-Commissives	10.1	43.2	56.8
**Interpersonal relations management**			
-Selfdisclosure	30.8	50.0	50.0
-Legitimize	14.7	98.9	1.1
-Reassure	12.9	92.2	7.8
-Joke	11.9	40.8	59.2
-Compliment	8.9	90.7	9.3
-Empathy	5.4	75.8	24.2
-Concern	4.8	86.2	13.8
-Smalltalk	3.8	21.7	78.2
-Compassion	3.0	100.0	0.0
-Comfort	2.7	93.8	6.2
-Appreciate	0.7	50.0	50.0
-Criticism	0.3	100.0	0.0

To understand intentions, processing steps and interaction strategies, semantic content information is required. We therefore enriched DA representations in DiAML with semantic content elements as described in Section 2.3, distinguishing different levels of detail. Table 4 shows the semantic content categories we used at three levels of specificity with their distribution. The first level corresponds to classes with sub-classes or attributes (characteristics or parameters that classes have) at the second and third level. As in an ontology, classes comprise concepts that are of certain type, sort, category, or kind. Classes may classify individuals, other classes, or a combination of both. For example, *medication* is a sort of *therapeutic regime, drugs* is a kind of *medication, pain killer* is a kind of *drugs*, and *co-codamol* and *lidocaine patches* are (instances of) *painkiller*, see also an example in (6). Instances, the basic or “ground level” objects (individuals), are annotated with the class label at any level of specificity, but as specific as possible. If there is serious doubt or not enough evidence about whether to choose a more or a less specific category, then the less specific one should be used since that tag subsumes the more specific class. Individuals are represented as values of an attribute which in turn can be a class or another individual, see example (6) below.

**Table 4:** Semantic content categories at multiple levels of specificity.

**1st level class**	**Functional**	**2nd level (sub-)class**	**Functional**	**3rd level (sub-)class**	**Functional**
	**segments (in %)**	**or attribute**	**segments (in %)**	**or attribute**	**segments (in %)**
medicalCondition	23.1	Symptoms	69.2		
				Anamnesis	14.2		
				Diagnosis	4.3		
				Prognosis	8.8		
				Other	3.5		
therapeuticRegimen	40.0	Tests	24.1	
				Medication	31.9	Use	13.7
								Interactions	0.8
								Dosage	35.9
								Effect	0.3
								Side-effects	13.4
								Precautions	0.6
								adverseReactions	0.6
								contrIndications	0.5
								Storage	0.6
								Guidelines	0.2
								Other	33.4
				Treatment	39.7		
				Other	36.2		
Feeling	4.0	Mood	27.1		
				Memories	0.5		
				Thoughts	11.3		
				Other	61.1		
lifeStyle	7.5	Occupation	35.3	Regimen	6.0
								Condition	9.0
								Other	75.0
				dailyActivities	0.5		
				habits	11.1		
				Diet	38.9		
				Sport	5.8		
				Clothing	0.5		
				Other	43.2		
socialCircumstances	5.5	Family	5.2		
				Partners	7.6		
				Friends	3.6		
				Employment	36.7		
				Other	46.9		
Admin	14.6	GPcontact	0.1		
				Prescription	22.5		
				Appointment	40.3		
				Forms	0.1		
				Arrangements	35.2		
				Other	1.8		
Services	5.2	collectMedication	24.8		
				Reminder	1.1		
				Other	5.7		
				Telemedicine	68.4	Technicalmeans	50.0
								channelQuality	19.8
								Other	30.2
Other	0.1				

**Figure 1:**
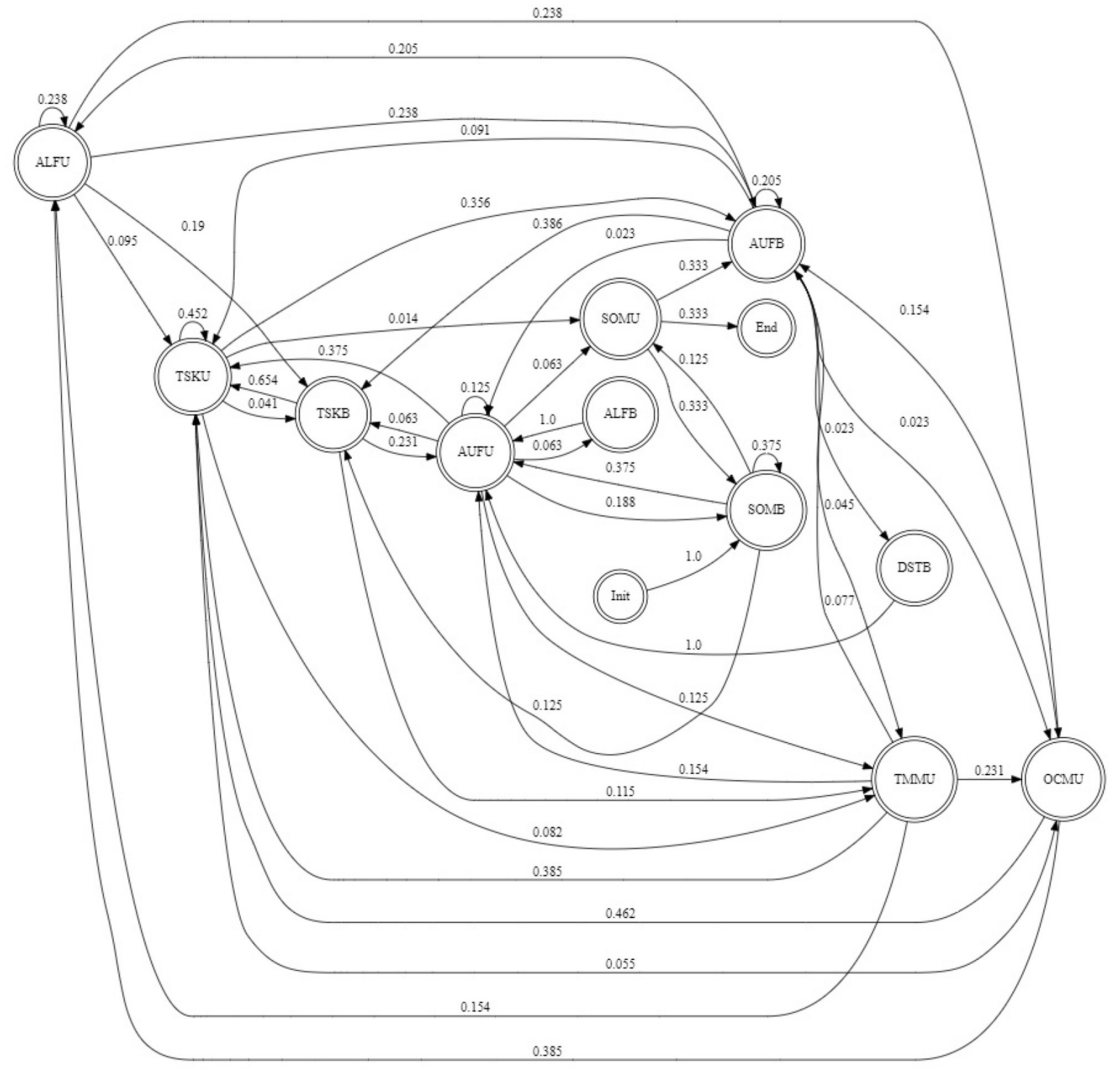
Probabilistic finite state automaton generated from ISO 24617-2 annotated VICO dialog data. SOM, Social Obligation Management; TSK, Task; AUF, Auto-Feedback; ALF, Allo Feedback; DST, Discourse Structuring; TRM, Turn Management; TMM, Time Management; OCM, Own Communication management; B, Bot; U, User.

We analyzed not only the distribution of dialog acts in the annotated dataset, but also their sequences to establish frequently occurring interaction patterns. Based on the observed DA co-occurrences and computed state transition probabilities, decision trees or finite state automata can be designed and used in the implementation of a conversational agent. Figure 1 illustrates the finite-state automaton generated from the ISO 24617-2 annotated data for the chatbot which is being designed to replace a doctor in medical consultations. The state transition graph represented here is based on dimension analysis focusing to define feedback and interaction management strategies, and not so much on task-related exchanges. The chatbot initiates the interaction by greeting the user, introducing itself and explaining its role and expertise. It will create opportunities for the user to provide feedback and/or return greetings, and introduce herself. In case of social acts like thanking, the user may reply either by giving feedback or accepting thanking, or both allowing multi-utterance turns. Multi-utterance turns were also expected from the user and generated by the chatbot combining Turn/Time Management and task-related actions, and in case of Own Communication Management acts which are followed by Stallings combined with Turn Keeping acts, and subsequent repeated or remained part of an interrupted task-related utterance. The chatbot frequently encourages the user to finish her utterance performing Auto-Feedback acts, e.g., generating backchannels and exhibiting active listening. In case user's stallings, corrections and disfluencies become persistent, occupying more than three consecutive segments, the chatbot may offer assistance in the form of an Allo-Feedback act or suggest to shift the topic. The full set of possible states and transitions between them incorporates information not only about dimensions, but also about communicative functions, qualifiers, and semantic content, potentially resulting in rich behavior and interactive strategies of a chatbot, and better understanding of how effectively partners talk to each other, how active each of them is, and how their communicative behaviors are interrelated.

### 3.2. Expert-assisted AI agent authoring

The creation from scratch of plausible dialog models that a conversational agent operates on, is challenging, time-consuming and requires considerable multidisciplinary expertise. Conversational agent designers who aim to develop not just a “chitchat” agent but an agent that is able to perform in a conversational way such tasks as searching databases and retrieving information, getting someone's support, or planning a therapy, lack the domain knowledge needed to perform in a semantically precise way. Domain experts have the necessary background knowledge to decide which information, resources and activities are important in which settings. They can share this information with agent designers, but would ideally rather make this information directly available to the agent. We developed an authoring tool to facilitate the (co-)creation of agents that are built using limited interactive data: they are supplied with initial, expert-authored state-action templates encoding domain knowledge, the agent's preferences concerning issues under discussion, expected outcomes, and decision-making strategies. The agent collects interactive experiences and learns from them. An example showcased in this paper concerns therapy planning negotiations.

**Figure 2:**
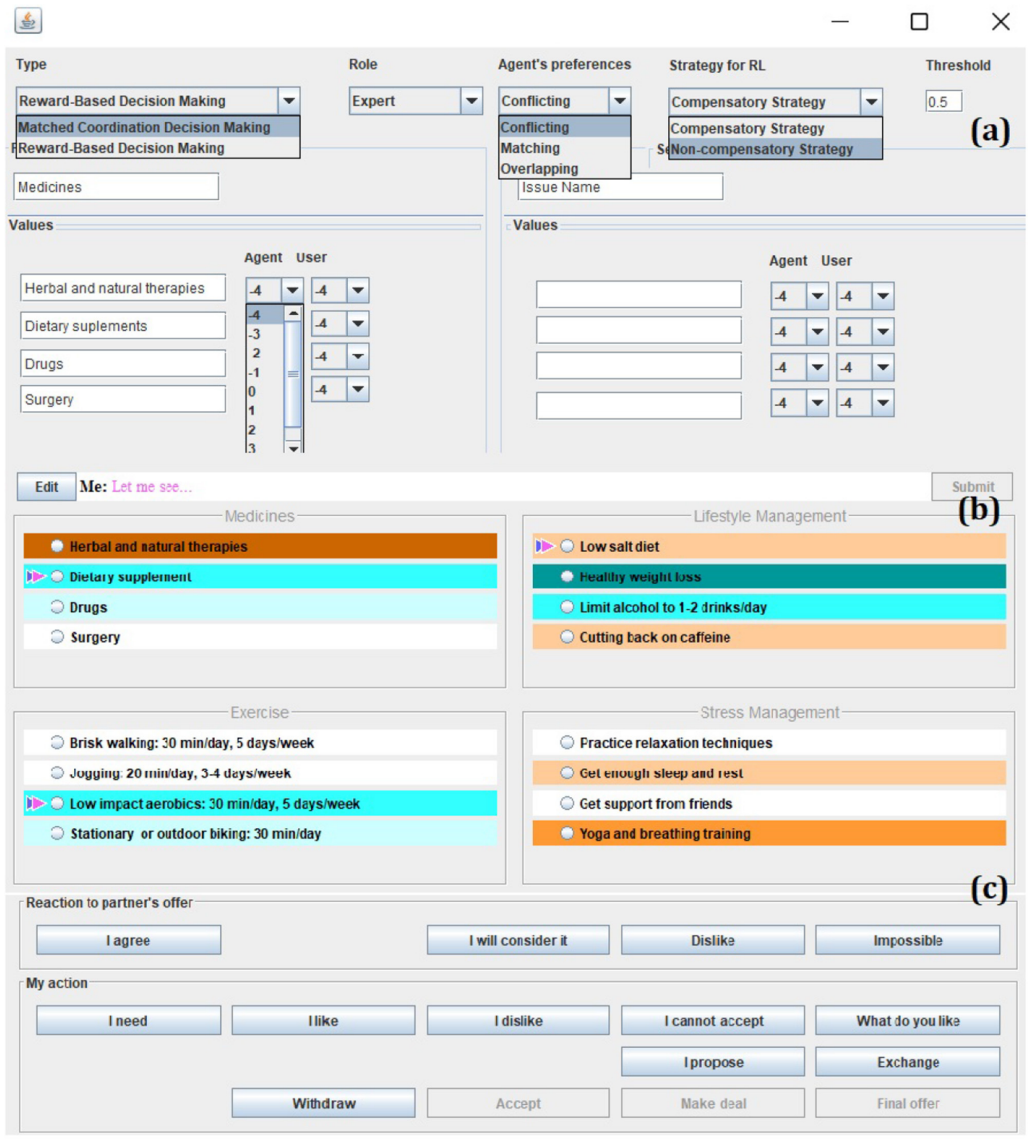
Authoring content and setting a preference profile for a “hypertension” scenario. From top to bottom: **(a)** menu to author negotiation values, setting a preference profile and negotiation strategy selection either by an expert or user; **(b)** resulted authored or automatically generated preference profile; and **(c)** an action selection menu.

We have developed a bargaining model where values are balanced such as the patient's best interest, modeled as the doctor's view on what is best for a patient, and patient autonomy - whether the patient is willing and able to adhere to a certain treatment (Petukhova et al., 2019). In negotiations, parties typically exchange offers expressing different levels of commitment, see Petukhova et al. (2017). Parties may propose trade-offs across issues in order for both sides to be satisfied with the outcome. Parties can postpone making an agreement or make a partial agreement on one issue, until the agreement on the second one is secured. They may withdraw from agreements during the interaction and revise their past offers, accept or decline any standing offer, and make counter-offers. Successful medical negotiation involves disclosure of preferences, and expression of the importance, desires and abilities concerning certain behavior and its outcomes. Table 5 provides an overview of actions used in negotiations. This action set has resulted from the interaction analysis discussed above, and presents a selection of the ISO 24617-2 dialog acts with extensions for Interaction Relations Management and semantic content specifications as provided by domain experts, see Figure 2.

**Table 5:** Taxonomy of the agent's actions.

**Interpersonal relations management**	**Task**	**Semantic content**		
		**Modality**	**Negotiation move**	**Issue(options)**
Compliment	(Open-ended) set question	Preference	(final) offer	Authored by domain
Empathy	(forced) choice question	ability	Exchange	experts or users
Compassion	propositional questions	necessity	Concession	
Concern/worry	check questions	acquiescence	deal	
Reassure/encourage	inform/answer		withdraw	
Legitimize	(dis-)agreement			
Self-disclosure	suggest			
Criticism	request/instruct			
	offer			

Adapted from the ISO 24617-2 dialog act taxonomy extended with the RIAS categories.

In the authoring tool we designed, the agent preference profiles can be set by domain experts or generated automatically, given the type of partner the human participant wants to negotiate with. An author can select from either identical, conflicting, matching, or overlapping preferences presented in the graphical user interface in colors: ranging from dark orange for highly dispreferred options to dark blue for highly preferred options, see Figure 2. Negotiators' preferences may be *identical, conflicting* negotiators' preferences are each other's opposites, *matching* preferences are of the same polarity, but different in strength, and *overlapping* preferences have some elements of the same polarity and strength.

An agent makes its decision to perform a certain action based on knowledge stored in its memory (ACT-R^4^ declarative memory) encoded in instances represented as traces of experiences (Petukhova et al., 2019). The ACT-R mechanisms allow to compute recent memory traces that are more likely to be retrieved, and the most frequent ones that have been created or retrieved more often in the past.

Authors can choose from three decision-making strategies: *matching coordination, compensatory*, and *non-compensatory*. All three strategies have been proven to be plausible in simulations of human decision making performance. The agent is trained to select between various alternatives under different contextual conditions with the goal to achieve acceptable negotiation outcomes (Petukhova et al., 2019).

After the agent has decided to perform a certain dialog act, a corresponding linguistic pattern is selected from a database to generate an utterance. Patterns are extracted from the VICO corpus and a negotiation corpus—the Multi-Issue Bargaining Corpus (MIB, Petukhova et al., 2016).^5^ Lexicalized patterns tagged with specific communicative functions were extracted and stored as templates with the variable fields for modalized semantic content as slots values. In total, 679 communicative functions patterns were extracted and 64 slot values specified. Although an initial set of patterns was rather small, the combination of sentence patterns with the ability to change individual values, allows the generation of utterances, broader than the target corpus (for comparison 5,781 MIB utterances resulted in 43,453 automatically generated utterances).

### 3.3. Interactive data collection with simulated conversational AI agents

Data-driven conversational agent design requires substantial amounts of data. Despite efforts that have been undertaken to build large data collections (see, e.g., Serban et al., 2017 for an overview), dialog researchers struggle with the aggregation of appropriate in-domain data of sufficiently high quality and in adequate amounts. The analysis of authentic interactions in real environments is generally expected to deliver the best understanding of natural human behavior, but authentic data collection is not only problematic for ethical reasons, but sometimes not even desirable, due to a lack of experimental control. For some use cases, restrictions need to be imposed to be able to investigate a controlled set of communicative activities and related phenomena without having to deal with unrelated details. Therefore, one may opt for specifically arranged forms of interaction such as *elicited interactions*, open and closed *role plays* and *simulations*. Such data collection methods can be effective for eliciting and examining authentic interactive behavior (Kasper, 2000; Bardovi-Harlig and Hartford, 2005). Role playing underpins simulations of communicative situations featuring real-life scenarios (Brône and Oben, 2015). Simulation with human actors or lay persons is often costly, and such data are not always accessible. Alternatively, simulated conversational agents can be used. It has been shown that even simple agents can exhibit rather complex emergent behavioral patterns (Hegselmann and Krause, 2002). Advanced agents can play the role of a believable human-like agent in various human-agent settings (Malchanau et al., 2019; Petukhova et al., 2019). In this section, we present the method of *interactive in-domain data collection*, using a relatively simple conversational agent called LICA^6^, developed by the expert-assisted approach discussed in the previous section. This activity again relies on the ISO 24617-2 data model and DA inventory.

Using the LICA tool we constructed the new LICA dialog corpus. Human-human and human-agent dialogs were collected. In the human-human setting, one participant was randomly assigned the role of a doctor, the other participant the role of a patient.^7^ The tool automatically generates preference profiles for scenarios of various complexity. The goal of each partner is to find out the preferences of the other and to search for the best possible mutual agreement. In human-agent negotiations, each human participant in the doctor's role negotiated with the simulated patient (the agent) who has different attitudes (preferences) and exhibits either cooperative or non-cooperative behavior, and uses different decision-making strategies. In total, we collected 25 human-human negotiations comprising 575 speaking turns, and 75 human-agent negotiations comprising 2,049 turns. Table 6 summarizes the core corpora properties.

**Table 6:** Comparison of human-human and human-agent negotiation performance in the LICA corpus.

**Evaluation criteria**	**Human vs. human**	**Human vs. agent**
Number of dialogs	25	75
Collection time (in min/per dialog)	9:40	3:50
Annotation time (in min/per min of dialog)	25	0.0
Mean dialog duration (in #turns)	23.0	21.3
Number of offers/per round	16.0	14.3
Dialogue Acts (# unique acts)	29	10
Vocabulary size (# unique tokens)	1864	517
Agreements (in %)	78.0	86.3
Pareto efficient agreement (in %)	82.4	90.3
Negative deals (in %)	21.0	34.3
Cooperativeness rate (in %)	39.0	61.9

The automatically generated dialogs are not rated as highly as human-human ones: human-agent dialogs do not have a rich vocabulary and the agent cannot deliver human-like interactive behavior, producing a rather scarce repertoire of dialog acts. Agents, however, show task-related negotiation and decision making behavior comparable to humans in terms of number of agreements reached and their Pareto efficiency, number of accepted negative deals, and cooperativeness rate. Provided with a set of profile parameters and a database of linguistic patterns, the tool instantly generates many full exchanges that are semantically annotated and evaluated. This reduces annotation time and costs significantly.

We envision immediate practical use of this method in the collection and exploration of behavioral and functional data. For example, relations between linguistic forms, dialog strategies and socio-pragmatic variability and their role in efficient decision making can be assessed in a controlled, systematic way. We have observed that human participants facing different types of agents used different negotiation tactics, resulting in different outcomes, such as delayed agreements, frequently revised offers, and adjustment of the alacrity to reveal or hide preferences. We have also noticed that participants of different gender or personality adopt different strategies under otherwise identical conditions. It may be concluded that our simple agents equipped with varying decision-making strategies offer plenty of opportunities to investigate relationships between a participant's intrinsic characteristics and a range of dependent variables characterizing their dialog behavior.

**Figure 3:**
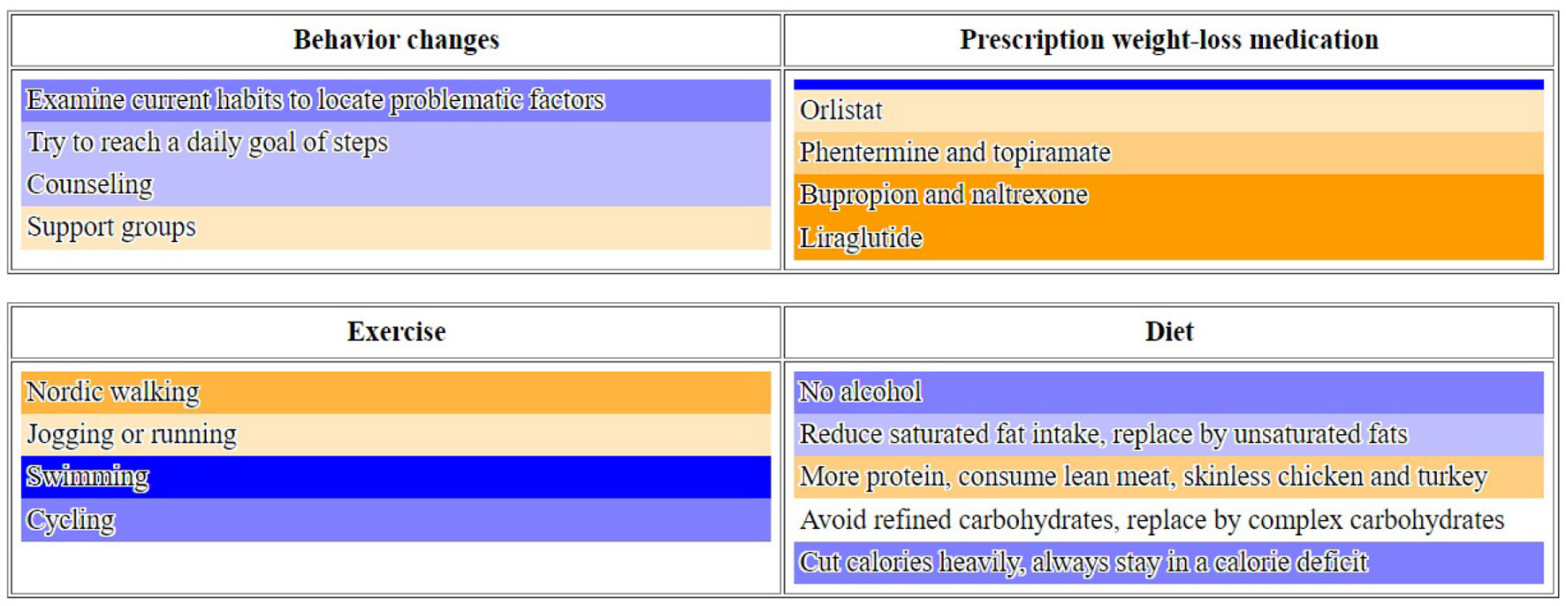
Example of a set participant's preference profile and action selection menu for “obesity” scenario.

To test the transferability across domains, we have used the tool for encoding domain knowledge for “obesity,” “diabetes,” and “smoking cessation” negotiation scenarios. Domain experts authored content from which preference profiles were generated. Figure 3 illustrates an “obesity” scenario. With little efforts, we collected a significant amount of semantically and pragmatically annotated data for training neural interaction models.

## 4. Refining ISO 24617-2 concepts for medical conversations

Functional and content descriptions of various degree of precision and detail, tailored to the application domain, open the perspective for more human-like interaction that is understandable and accepted by domain experts and their users, staying close to their reality and process models. The analytical and empirical studies reported in the previous section showed what functional and semantic content aspects of dialog act specifications in DiAML need extensions in order to facilitate semantically and pragmatically adequate analysis of interactive behavior and to optimally support the design of intelligent conversational agents. In this section we summarize these extensions and formalize their DiAML representations. Functional aspects concern the enhanced set of *communicative functions* and their possible *qualifiers*. Semantic content aspects encompass the augmented set of *dimensions* and the articulation of *semantic content*.

### 4.1. Semantic content aspects

Our studies show that the addition of the dimension of *Interpersonal Relations Management* (IRM) leads to a pragmatically more precise analysis and modeling of those communicative actions in the medical domain which are meant to establish a certain bond between the dialog participants. Successful partnership building actions promote better cooperation. In medical consultations, actions are important that convey the doctor's alliance with the patient in terms of health and support, decision-making, or the development of a therapeutic plan.

RIAS supports a high-level specification of the semantic content of medical actions. Task-focused actions are about *medical conditions, therapeutic regime, lifestyle, psychological feelings, services, medication*, and *other* content. An alternative medical interaction analysis system, Medical Interaction Process System (MIPS, Ford et al., 2000), defines additional semantic content categories, such as *tests, side-effects, drugs, social/demographic circumstances*, and *administrative/practical details*. To model domain-specific semantic content with adequate detail and leave room for future improvements and extensions, we specified semantic content at various levels of detail, where broad categories comprise a number of more fine-grained classes (Table 4).

A negotiation-specific semantics, as discussed in Petukhova et al. (2017), comprises specifications of “negotiation moves” such as offer, counter-offer, and concession, and their arguments. The  < NegotiationSemantics> element has been added to DiAML for representing the semantic content of a task-specific dialog act in a negotiation dialog. This element uses a  < NegotiationMove> element with attributes defined for different types of such moves. The  < avContent> element specifies domain-specific semantic information in terms of attribute-value pairs, and the  < modalLink> element (of type preferences, priorities, needs, and ability) links a @holder to the semantic content, as discussed in Lapina and Petukhova (2017). The example in (6) illustrates this. Speaker P2 *requests* “lidocaine patches” as substitute for an other *painkiller medication*, “co-codamol,” and by this performs a negotiation move of *counter-offer*.

(6) P1: It is quite safe to take co-codamolP2: Could I get some more of these lidocaine patches?

< dialogAct xml:id="dap1TSK38" sender="#p2" addressee="#p1"dimension="task" communicativeFunction="request"target="#fsp1TSKCV38"> < NegotiationSemantics> < NegotiationMove xml:id="nm33" type="counterOffer"/ > < avContent xml:id="av33" target="#nm33"attribute="therapeuticRegimen:medication:painkiller"value="lidocainePatches"/ > < modalLink holder="#p1" target="#av33"modalRel="preference"/ > < /NegotiationSemantics> < rhetoricalLink rhetoAntecedent="#dap2TSK37"rhetoRel="substitution"/ > < /dialogAct>

### 4.2. Functional aspects

A set of dimension-specific communicative functions has been defined for dialog acts in the IRM dimension, see Table 3. These actions are used to communicate interest, respect, support and empathy and to recognize and resolve relational barriers. Expressions of worry and concern, comfort, reassurance, compliment and compassion are more frequently produced by the doctor, e.g., “You shouldn't feel bad if you can't do the physical stuff,” “You are doing great, things will continue to improve in that way,” “We'll support you and get you through it”. Partnership-building actions like social talk and jokes are also used by the patient, e.g., “I won't tell you the rest of the story because it's too awful for words [laugh],” “It was worth a try [laugh],” “Can I get a new body [laugh]?”. If the doctor succeeds, patients reveal personal information about themselves, which can include thoughts, feelings, aspirations, goals, failures, successes, fears, and dreams, as well as what are one's likes or dislikes, e.g., “It's something that I'm particularly concerned about,” “I'll tell you what I would like if you don't mind,” “Even the slightest little thing is just setting me off and there's no reason for it,”

Two dimension-specific communicative functions for Task Management were frequently observed in VICO medical consultations: (1) *Give Orientation* for statements and directives related to an examination or clinical visit, e.g., “This is the telephone follow-up consultation,” “Just to remind you we're going to be recording the consultation”; and (2) *Discuss Expertise* related to participant roles and areas of expertise, e.g., “I am your cardiologist,” “The doctor who's organizing things with the study had explained it well”. General-purpose communicative functions were often used for therapeutic regime management and administrative arrangements, e.g., “I saw you a couple of weeks ago” (CheckQuestion), “I'll check with you at the end that you're happy for us to pass on the recording to the people who are doing the study as well” (Promise).

Question-answering parts are pervasive in medical encounters, for instance for medical history taking and clarification of complaints. RIAS differentiates between more focused questions (“*closed-ended”*) and more open questions (“*open-ended”*) that allow greater respondent discretion and a more detailed response. In our annotation experiments, annotation of question forms was found to be complicated but important. It has for instance been observed that a medical consultation should preferably start with an open-ended question, which conveys an interest in listening, whereas an early pursuit of closed questioning may prevent the discovery of all relevant issues, and may even lead to an incorrect diagnosis. RIAS suggests that closed-ended questions produce focused and curtailed responses, while open-ended questions are indicative of exploratory, investigative or unspecific probing. Questions where the speaker wants to obtain the truth of a proposition or wants to know some or all of the elements of a certain set, thus requiring a specific answer, are closed-ended questions. An open-ended question, as the name suggests, does not seek a specific answer at all.

To shape patients' responses, doctors often ask questions while suggesting one possible answer, e.g., by focused choice, leading, or confirmatory questions, or questions that carry certain assumptions and invite (dis-)agreement (some of which are defined in the DIT++ taxonomy^8^), openness or evasion, and threat or comfort. The ISO 24617-2:2012 set of question types can be extended to model these differences as special cases of propositional questions, set questions, choice questions, and check questions.

Medical encounters also involve counseling, where doctors *direct* the behavior of their patients, expressing the wish that the patient performs or avoids certain actions. Different types of directives carry different strengths of the speaker's assumptions about the ability and willingness of the addressee to perform (or avoid) an action. As noticed above, *commissive* acts play an important role in medical negotiations. For adequate modeling, we need to take into account that participants may perform several types of dialog acts expressing various *levels of commitment*, but also qualified actions expressing participants' attitudes and preferences, and negotiation strategies.

A doctor must be aware of a patient's feelings, motivations, insecurities, engagement and reasons for whether the patient wants to do certain things or not. In ISO 24617-2 this information can be annotated using sentiment qualifiers for which the standard does not specify a particular set of tags. RIAS defines a set of *global affects* that can be used in an ISO 24617-2 plug-in for the specification of participants' attitudes such as six binary categories for anxiety/nervousness, depression/sadness, emotional distress/upset, dominance/ assertiveness, interest/ attentiveness, friendliness/warmth. To continuously assess/measure emotions in dialog and to reliably compute *affective states*, Russell's circumplex model of affect (Russell, 1980) can be adopted, enriched with the Verona Coding Definition of Emotional Sequences (VR-CoDES), specifically defined for the domain of medical communication. The categories comprise 12 qualifiers such as *alert, excited, calm, nervous, afraid*, and *stressed out*, clustered with respect to emotional valence (positive vs. negative) and arousal (high vs. low).

## 5. Discussion and conclusions

Future intelligent conversational agents will operate on huge, dynamic, heterogeneous data streams, providing powerful possibilities for adaptive and flexible interaction. Existing conversational agents became successful and robust due to the sheer amount of real user data available to their developers. Nevertheless, these systems still exhibit rather restricted communicative behavior modeled on information seeking tasks. Conversational agents developed for research purposes allow for more natural conversations, but they are often restricted to a narrow, manually crafted domain. The most recent trend in conversational agent design incorporates neural networks and transformer models, trained on huge collections of dialog data without a detailed specification of dialog states. These models lack controllability and interpretability due to their black-box nature. They also require extensive supervised training data in order to perform competitively. Thus, the sheer amount of available data does not automatically lead to agent behavior that can be acknowledged as intelligent; to embed intelligence into data-driven dialog modeling and agent design requires appropriate and adaptable inventories of semantic concepts and skilled/expert manual annotation work.

The study presented in this paper addresses the above mentioned issues. The data-driven analytical methodology that we proposed to design and test intelligent conversational agents relies on rich semantic and pragmatic annotations of authentic conversational data, and can achieve a high precision in description and modeling of intelligent interactive behavior. The main building blocks of our model are representations of the information states of dialog participants (agents and humans) and an inventory of actions they may perform. Existing dialog act annotation frameworks, like DAMSL, SWBD-DAMSL, and MIDAS annotate only the functional aspects of dialog utterance meanings, essentially what are the communicative functions of an utterance, DIT++ and the ISO 24617-2 standard derived from it are the first frameworks that specify additional DA information, namely semantic dependence relations, pragmatic (“rhetorical”) relations, qualifiers which fine-tune a communicative function, and the semantic content of an utterance.

**Figure 4:**
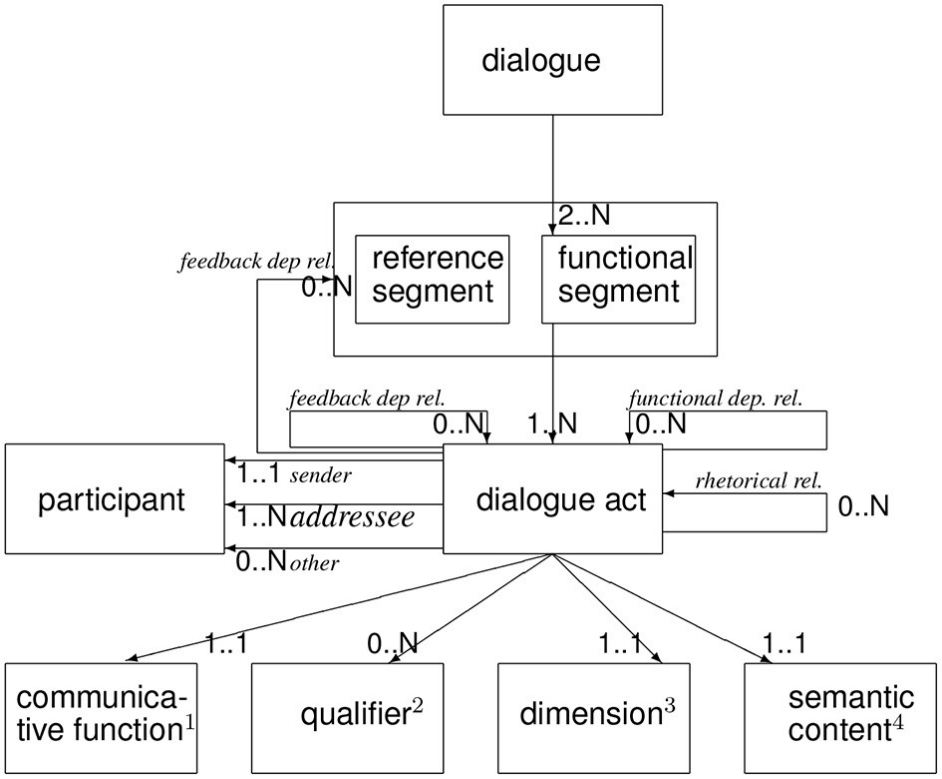
ISO 24617-2 Extended Metamodel: (1) with communicative functions for task-specific DAs and dimension-specific functions for Task Management and IRM. (2) with additional qualifiers for emotions. (3) with additional dimension: Interpersonal Relations Management (IRM). (4) plug-in for articulate semantic content representation.

The ISO 24617-2 data model (or “metamodel”) captures the way these categories of information are related, see Figure 3, which shows the upper-level concepts involved in DA annotation or specification. Note that the functional segments that make up a dialog each express one or more dialog acts, reflecting the multifunctionality that utterance segments often display (Bunt, 2011). Each dialog act has a semantic content of a certain general type (“dimension”), an articulate semantic content represented by a domain-specific plug-in, a communicative function, and possibly a number of qualifiers. DiAML was designed to represent these information categories, and the present study shows that this can be done in a pragmatically and semantically precise way for the domain of medical consultation by extending the ISO data model in four respects, corresponding to the four boxes at the base of Figure 4:

1. Dimensions: add the dimension Interpersonal Relations Management (IRM).
2. Communicative functions: add a distinction between open-ended and closed-ended (from RIAS) to the general-purpose functions, and add the following dimension-specific functions:

- For the Task Management dimension: Give Orientation and Discuss Expertise
- For the IRM dimension: SelfDisclosure, Legitimize, Reassure, and 9 more—see Table 3.

3. Qualifiers: attributes and values for expressing affect (emotions, feelings, mood, attitudes, sentiment) such as anger, anxiety, distress, responsiveness, and respectfulness—see discussion above and Petukhova and Bunt (2020), Table 4.
4. Semantic content: a plug-in as outlined in Section 2 and illustrated in 6—for more detail see Bunt (2019).

These extended conceptual inventories and tools make it possible to achieve high levels of semantic and pragmatic precision in dialog analysis and modeling, from which a conversational agent may benefit to boost the intelligence of its behavior.

The extensions were defined in a series of analytical and empirical experiments targeting three important use cases: (1) interaction analysis for agent design; (2) expert-assisted and user-driven AI agent authoring; and (3) interactive in-domain annotated data generation. The extended ISO 24617-2 repository of communicative functions and the semantically enriched DiAML representations have proven to facilitate these use cases across various domains.

We envision significant practical and theoretical use of the proposed method for adequate modeling and data-driven design of a new generation of intelligent conversational agents. The proposed systematic and comprehensive interaction analysis and dialog modeling method can be used for obtaining a satisfactory degree of understanding of human interactive behavior, is a basis for the specification of mechanisms of human dialog to be incorporated into an intelligent dialog system. Particular challenges for future work are the modulation of various semantic and pragmatic precision aspects in experimental agents in order to systematically investigate factors influencing interaction outcomes, and the testing and modification of a range of successful and failed interactive strategies.

## Data availability statement

The original contributions presented in the study are included in the article, further inquiries can be directed to the corresponding author.

## Ethics statement

Ethical approval was not required for the study involving human participants in accordance with the local legislation and institutional requirements. The participants provided written informed consent to participate in this study.

## Author contributions

Both authors listed have made a substantial, direct, and intellectual contribution to the work and approved it for publication.

## Footnotes

^1^Note that DiAML uses standoff annotation, where the value of the attribute “target” is a markable that anchors the annotation in the source text.

^2^See Bunt et al., 2019 for the use of alternative representations of DIAML annotations.

^3^ https://www.ed.ac.uk/usher/telescot/projects/completed-projects/vico

^4^A Java Simulation and Development Environment for the ACT-R was used; http://cog.cs.drexel.edu/act-r/about.php.

^5^The MIB corpus is released in the Linguistic Data Consortium catalog under the reference number LDC2017S11; https://catalog.ldc.upenn.edu/LDC2017S11.

^6^LICA stands for Learning Intelligent Conversational Agent.

^7^Human participants were educated about the purpose and the course of the study. Subsequently, a declaration of informed consent is signed. The anonymity of the participants is guaranteed followed the European laws on personal data protection (GDPR, see https://gdpr-info.eu/).

^8^See https://dit.uvt.nl.
